# Bleeding

**Published:** 1879-08

**Authors:** 


BLEEDING.

In severe cases, the only thing that can be depended upon is pressure. Press your thumb or finger on or into the bleeding place and keep it there till you can have assistance.

If the bleeding is below the middle of the thigh, tie a cravat or pocket-handkerchief around the thigh, some distance above the wound, then put a stick under the handkerchief and twist until the bleeding stops.

A more simple way is to press the thumb on the artery near the wound, and between it and the heart, till the bleeding stops. Then keep up the pressure until the physician comes. The great danger in these cases is that the person upon whose skill and coolness a life may depend, occasionally finds his efforts paralyzed by fear and anxiety.

Nelaton said to his class of medical students, "If you should by accident sever the carotid artery the patient would bleed to death in four minutes, *therefore there is no time to be in a hurry*."

In all accidents and emergencies the road to safety is in coolness and self-possession. It is of more value than skill without it.

